# Additional data for the mouse TRPV6 expression atlas

**DOI:** 10.1016/j.dib.2022.108201

**Published:** 2022-04-22

**Authors:** Philipp Wartenberg, Femke Lux, Kai Busch, Claudia Fecher-Trost, Veit Flockerzi, Gabriela Krasteva-Christ, Ulrich Boehm, Petra Weissgerber

**Affiliations:** a Department of Experimental and Clinical Pharmacology and Toxicology, Center for Molecular Signaling (PZMS), Saarland University School of Medicine, Homburg, Germany; b Institute of Anatomy and Cell Biology, Saarland University School of Medicine, Homburg, Germany

**Keywords:** TRP channels, Calcium, Genetic labeling, Expression

## Abstract

To identify TRPV6 expression in the whole mouse with a cellular resolution we took advantage of TRPV6-IRES-Cre knock-in mice crossed with the enhanced ROSA26-τGFP reporter line. In the resulting TRPV6-IC/eR26-τGFP animals, TRPV6-expressing cells are labeled with τGFP. Data were collected from organs prepared from fixed experimental adult and juvenile TRPV6-IC/eR26τGFP and Cre-negative eR26-τGFP control animals of both sexes. Organ cryosections from each age and sex were stained for GFP and imaged with a slide scanner. Here, we describe reporter gene expression in a large number of tissues. We also document the absence of τGFP signal in the corresponding Cre-negative control tissues, including controls for the TRPV6 expression data described in [1]. The data reported here and in [1] constitute the TRPV6 expression atlas for the mouse. Our data offer a wealth of information to enable investigation of the functional role of TRPV6 channels in different tissues.

## Specifications Table

**Table:** 

Subject	Molecular Biology
Specific subject area	Mapping of TRPV6 expression in the mouse with cellular resolution by analyzing fluorescently labeled cryosections acquired from TRPV6 reporter mice and comparing them to control tissues.
Type of data	Image Figure
How the data were acquired	Data were acquired from organs dissected from adult and juvenile TRPV6-IRES-Cre/eROSA26-τGFP animals of both sexes and Cre-negative eROSA26-τGFP controls. Images of GFP-stained sections from these organs were imaged with an AxioScan slide scanner (Zeiss) using the ZenBlue software.
Data format	Analyzed
Parameters for data collection	Data were collected from GFP-stained cryosections prepared from fixed mouse organs of experimental adult and juvenile TRPV6-IRES-Cre/eROSA26-τGFP and Cre-negative eROSA26-τGFP control animals of both sexes.
Description of data collection	Experimental adult and juvenile TRPV6-IRES-Cre/eROSA26-τGFP and eROSA26-τGFP control animals of both sexes were transcardially perfused with 4% paraformaldehyde and organs were dissected. Cryosections of organs were stained with an antiserum against GFP and imaged with the AxioScan slidescanner from Zeiss with ZenBlue software.
Data source location	Department of Experimental and Clinical Pharmacology and Toxicology, Center for Molecular Signaling (PZMS), Saarland University School of Medicine, Homburg, Germany.
Data accessibility	Repository name: Zenodo Data identification number: 10.5281/zenodo.6341244 Direct link to the dataset: https://zenodo.org/record/6341244#.Yk2l2jVCQuV
Related research article	P. Wartenberg, F. Lux, K. Busch, C. Fecher-Trost, V. Flockerzi, G. Krasteva-Christ, U. Boehm, P. Weissgerber, A TRPV6 expression atlas for the mouse, Cell Calcium, 100 (2021) 102481.

## Value of the Data

• Generation of reporter mouse strains will help understanding gene expression in an organism-wide manner with a single cell resolution.
• Data provide an unprecedented wealth of information to investigate the physiological function of defined genes in individual cells/organs *in vivo*.
• Expression atlas can be used by other researchers to identify TRPV6-expressing cell types in different organs of interest
• Data can be used as a reference for further investigation including primary cell isolation

## 1 Data Description

Data were collected from adult and juvenile TRPV6-IC/eROSA26-τGFP male and female reporter (Cre+) and control animals (Cre-). If not otherwise stated, we did not detect any gross gender or age differences.

**Fig. 1:**
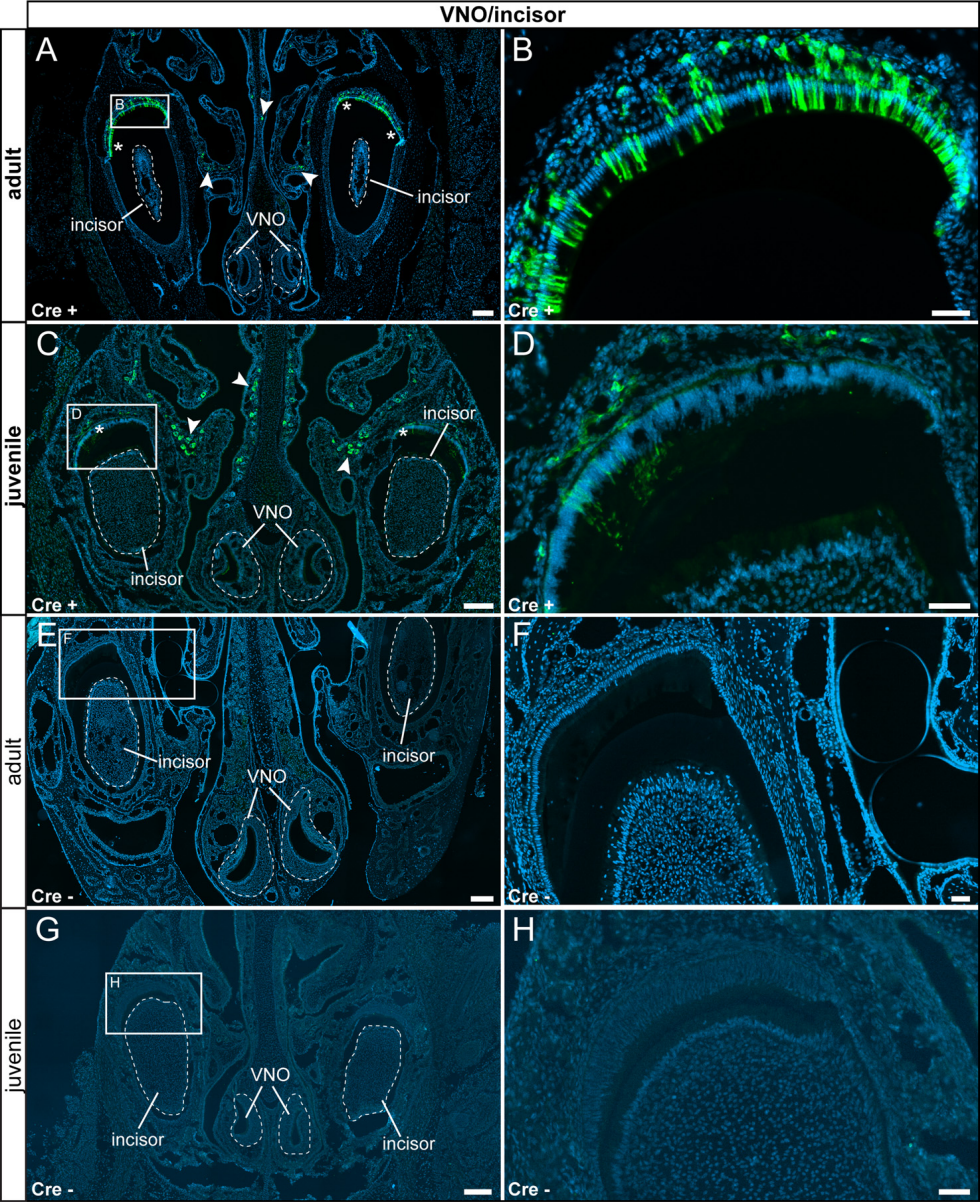
Reporter gene expression was found in enamel-secreting epithelial cells and a subset of mucus-secreting cells in the olfactory epithelium. (A) In adult experimental animals (Cre+), a subset of high prismatic epithelial cells, located in the epithelial cell layer adjacent to the incisors (asterisk), expressed τGFP. Please note the absence of τGFP in the incisors, demonstrating a lack of TRPV6 in teeth. In addition, τGFP was also found in mucus-secreting cells (arrowheads) in the olfactory epithelium. The vomeronasal organ (VNO) did not show τGFP expression. (B) Magnified image of the area indicated in (A). (C) A similar pattern was observed in juvenile Cre+ animals, but the number of high prismatic epithelial cells was lower. (D) Magnified image of the area indicated in (C). (E) We did not detect any reporter gene expression in adult control tissues (Cre-). (F) Magnified image of area indicated in (E). (G) We did not detect any reporter gene expression in juvenile control tissues (Cre-). (H) Magnified image of the area indicated in (G). Nuclear stain in blue. Scale bars: 200 µm (A, C, E and G), 50 µm (B, D, F and H).

**Fig. 2:**
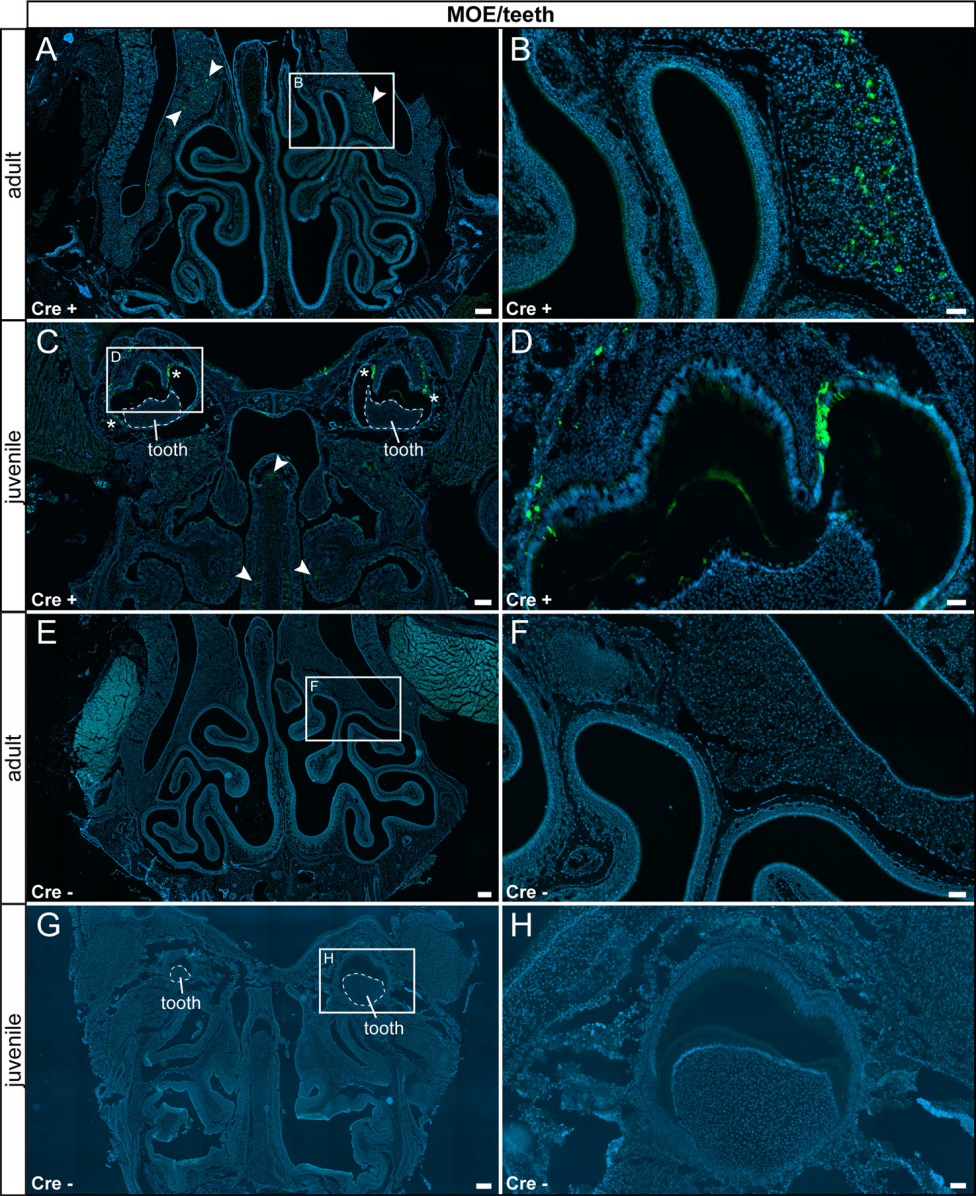
τGFP expression was observed in mucus-secreting cells below the main olfactory epithelium (MOE). (A) In the adult MOE reporter gene-expressing cells were found in mucus-secreting cells below the olfactory epithelium in Cre+ animals (arrowheads). (B) Magnified image of area indicated in (A). (C) In juvenile Cre+ animals, a similar pattern was observed. Please note the τGFP-expressing subset of the enamel-secreting cells (asterisk). (D) Magnified image of the area indicated in (C). (E) Reporter gene expression was not detected in adult Cre- control animals. (F) Magnified image of area indicated in (E). (G) No reporter gene expression was observed in juvenile Cre- controls. (H) Magnified image of the area indicated in (G). Nuclear stain in blue. Scale bars: 200 µm (A, C, E and G), 50 µm (B, D, F and H).

Beginning with the nose, we did neither detect reporter gene expression in sensory neurons of the vomeronasal organ (VNO) (Fig. 1 A–D) nor the main olfactory epithelium (MOE) (Fig. 2 A–D). Below the olfactory epithelium, we identified reporter gene-expressing cells in the mucosa (Fig. 1 A–D and Fig. 2 A–D). Next to the olfactory epithelium, we found high prismatic τGFP+ cells in the enamel-secreting cell population (Figs. 1 and 2). Please note that we detected more τGFP cells in adult animals in the epithelial cell layer next to the incisors when compared to juvenile animals. The observed difference in cell number reflects the lower number of cells showing TRPV6 promotor activity indicating reduced a need for Ca^2+^ signals in these cells during early development. We did not detect any reporter gene expression in control tissues (Fig. 1 E–H, Fig. 2 E–H).

**Fig. 3:**
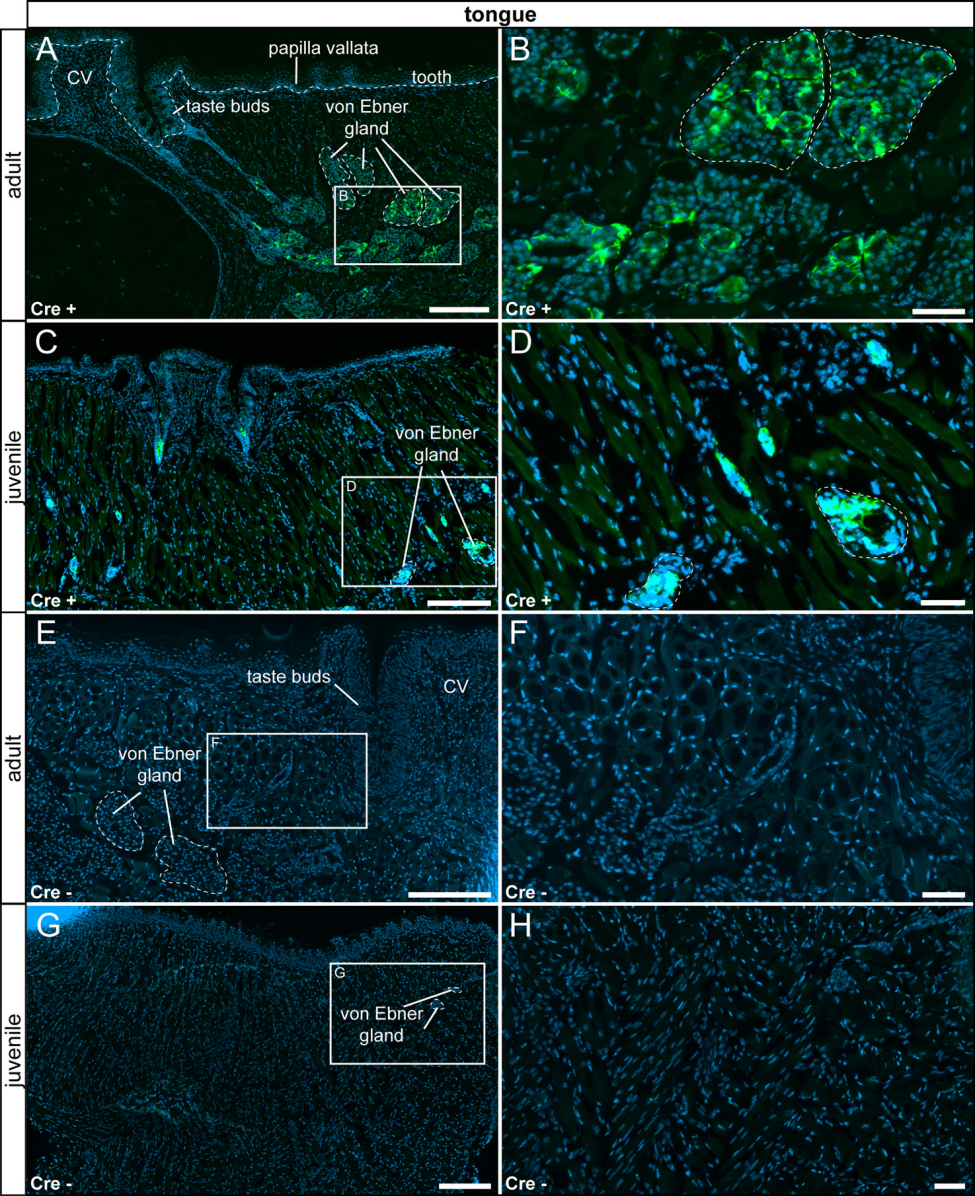
τGFP-expressing cells within the tongue of experimental (Cre+) and control (Cre-) animals. (A) In the adult tongue, τGFP+ cells were found in a subset of cells forming the mucous glands (von Ebner gland) in Cre+ animals. (B) Magnified image of the area indicated in (A). (C) A similar reporter expression pattern was observed in juvenile Cre+ animals, however with fewer τGFP+ cells. (D) Magnified image of the area indicated in (C). (E) No reporter gene expression was found in adult Cre-controls. (F) Magnified image of the area indicated in (E). (G) Reporter gene expression was not detected in juvenile Cre- controls. (H) Magnified image of area indicated in (G). Nuclear stain in blue. CV: circumvallate papilla. Scalebars: 200 µm (A, C, E and G), 50 µm (B, D, F and H).

In the tongue's *lamina propria*, we identified reporter gene expression in mucous and serous glands (Fig. 3 A–D). We did not detect τGFP signal in the tongue of control animals (Fig. 3 E - F).

**Fig. 4:**
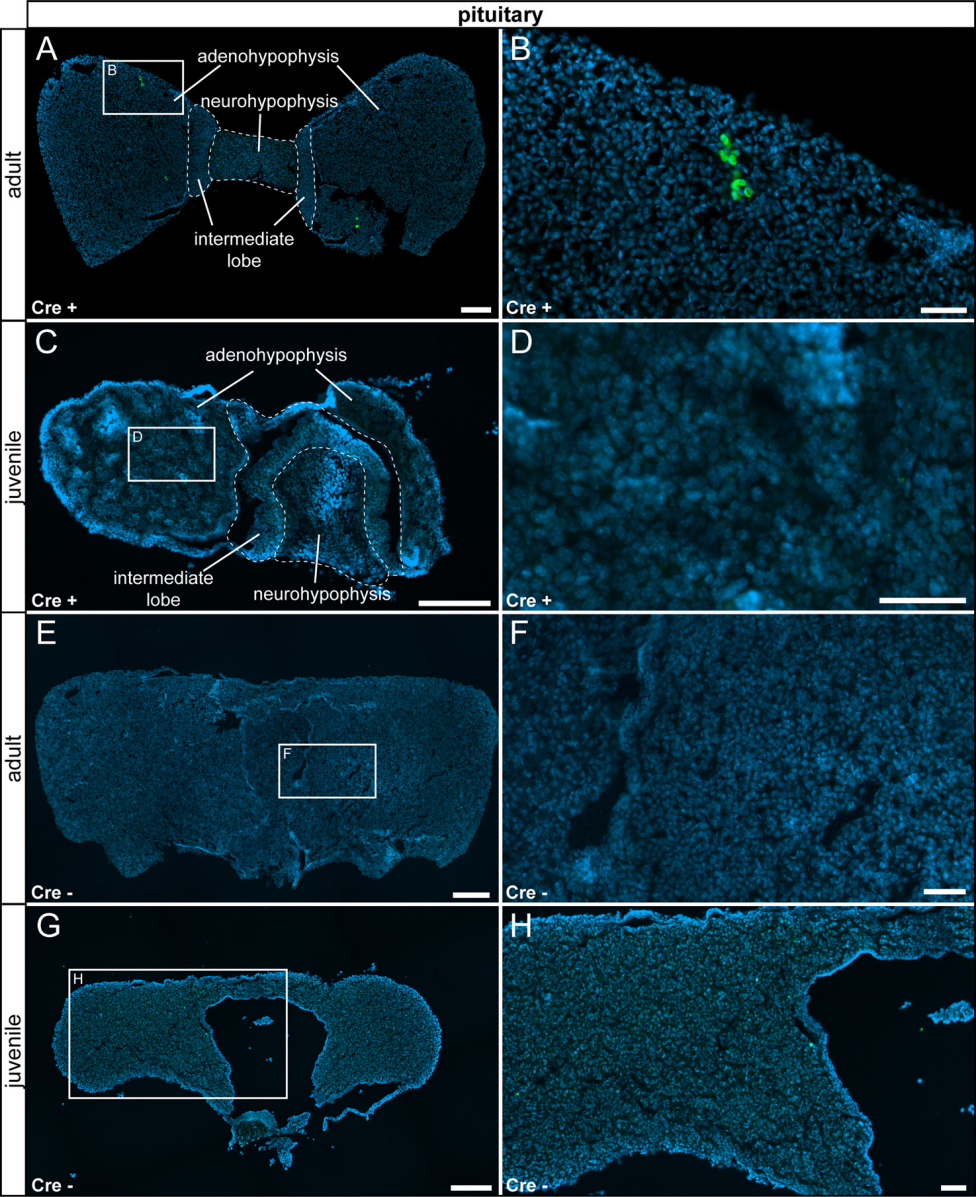
TRPV6 expression in the pituitary gland is restricted to adult animals. (A) Few τGFP+ cells were found in the anterior pituitary of adult Cre+ animals. (B) Magnified image of the area indicated in (A). (C) No reporter gene expression was observed in juvenile Cre+ animals. (D) Magnified image of area indicated in (C). (E) Reporter gene expression was absent in the adult Cre- pituitary. (F) Magnified image of area indicated in (E). (G) No reporter gene expression was observed in juvenile controls (Cre-). (H) Magnified image of area indicated in (G). Nuclear stain in blue. Scalebars: 200 µm (A, C, E and G), 50 µm (B, D, F and H).

**Fig. 5:**
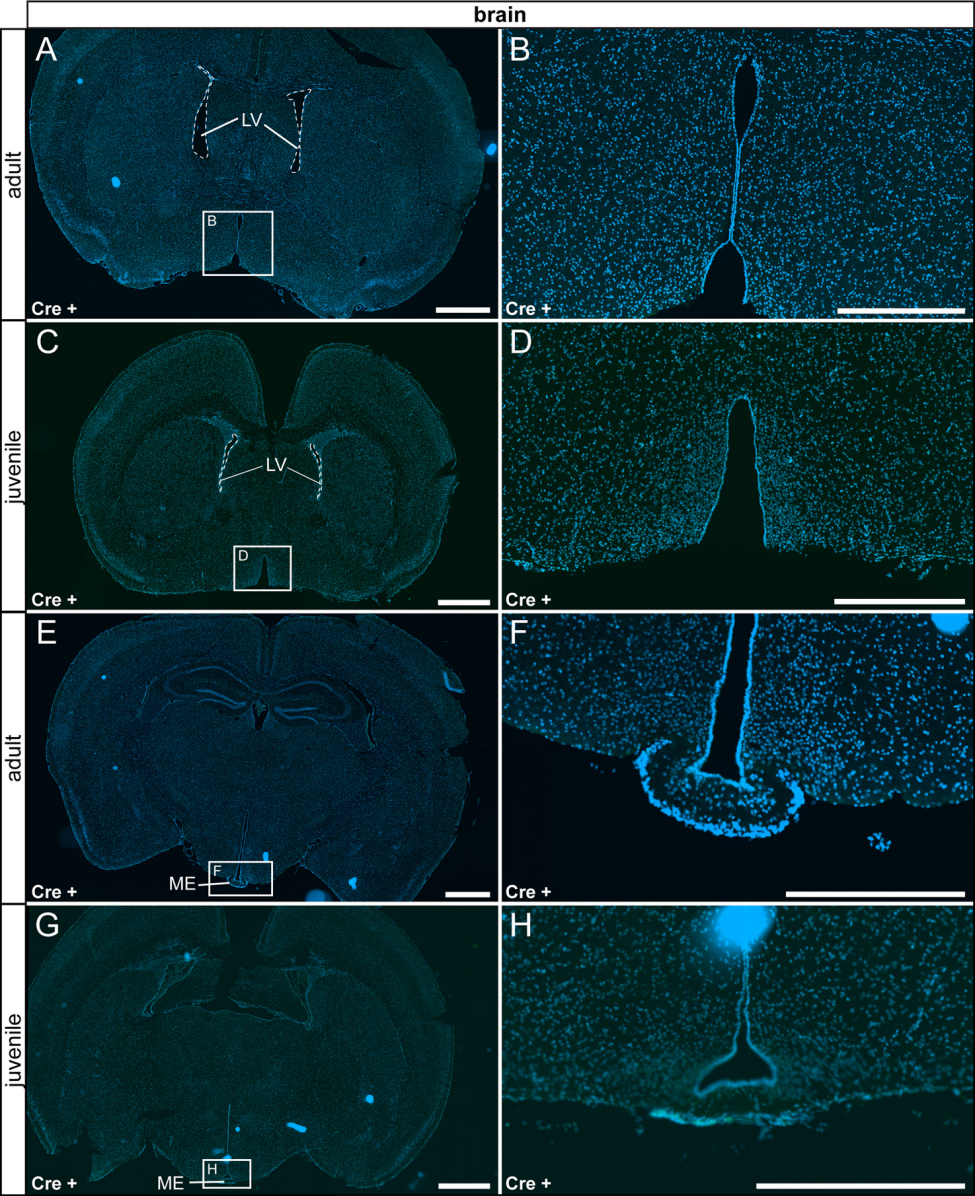
TRPV6 is not expressed in the brain. (A) We did not detect τGFP expression in the adult reporter brain. Shown is an example picture from rostral hypothalamus. (B) Magnified image of the area indicated in (A). (C) The same was observed in Cre+ juvenile animals, shown is a similar region as in (A). (D) Magnified image of the area indicated in (C). (E) Shown is a more caudal region of the hypothalamus containing the arcuate nucleus and the median eminence (ME) from adult Cre+ animals. No reporter gene expression was observed. (F) Magnified image of the area indicated in (E). (G) Reporter gene expression was also absent in more caudal hypothalamic regions in juvenile Cre+ animals. (H) Magnified image of the area indicated in (G). LV: lateral ventricle. Nuclear stain in blue. Scale bars: 1000 µm (A, C, E and G), 500 µm (B, D, F and H).

**Fig. 6:**
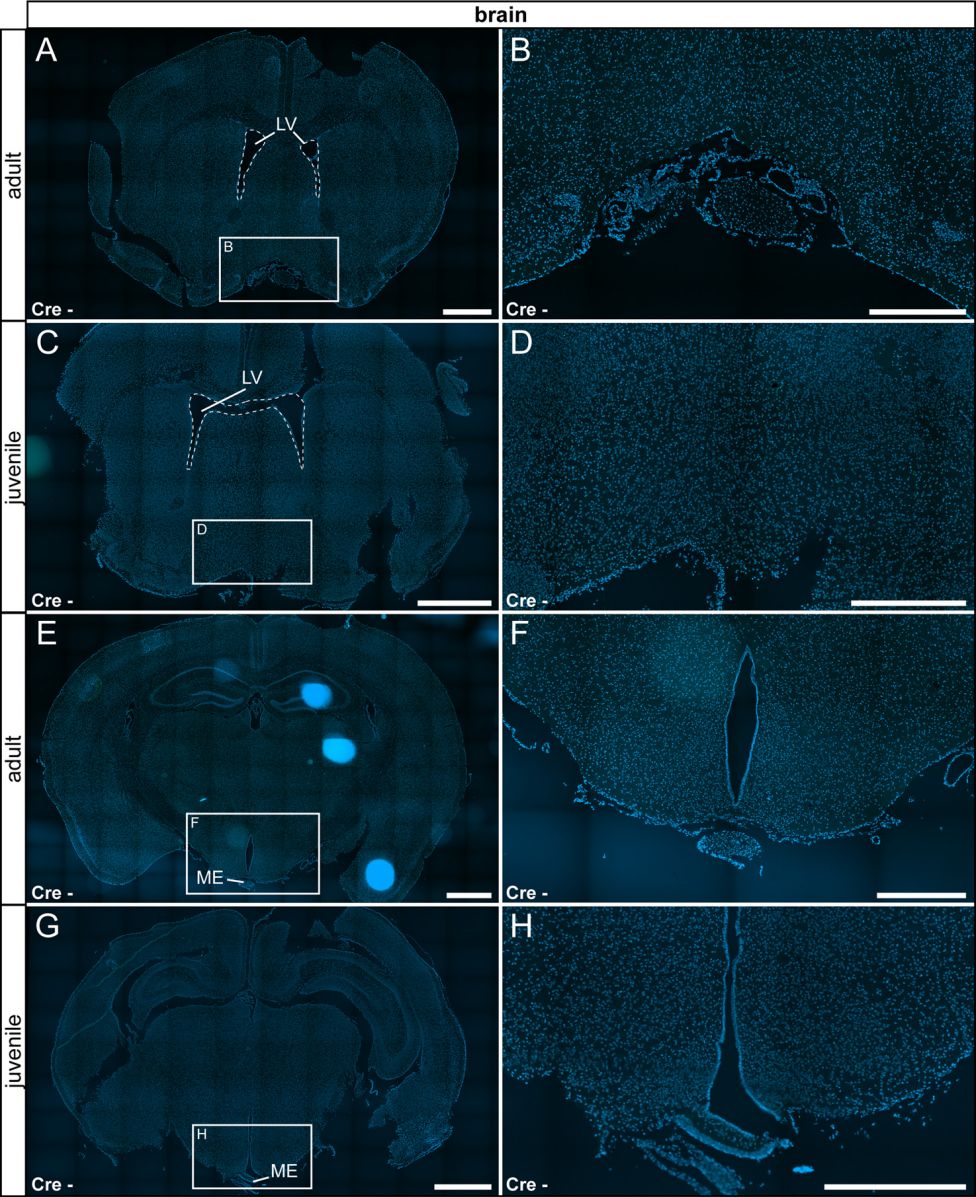
No reporter signal in the brain prepared from control animals (Cre-). (A) Shown is an area from the rostral hypothalamus in adult controls (Cre-). (B) Magnified image of the area indicated in (A). (C) Similar region as in (A) is shown for juvenile animals. (D) Magnified image of the area indicated in (C). (E) Shown is a hypothalamic region including the arcuate nucleus and the median eminence (ME). No reporter gene expression was found in adult animals. (F) Magnified image of the area indicated in (E). (G) Similar region as in (E) in juvenile animals. (H) Magnified image of the area indicated in (G). LV: lateral ventricle. Nuclear stain in blue. Scale bars: 1000 µm (A, C, E and G), 500 µm (B, D, F and H).

In the nervous system, we detected only very few reporter gene expressing cells in the pituitary gland, but no τGFP cells were observed throughout the whole brain of both adult and juvenile animals (Fig. 4 A–D, Fig. 5). Control tissue was devoid of reporter gene expression (Fig. 4 E - F, Fig. 6).

**Fig. 7:**
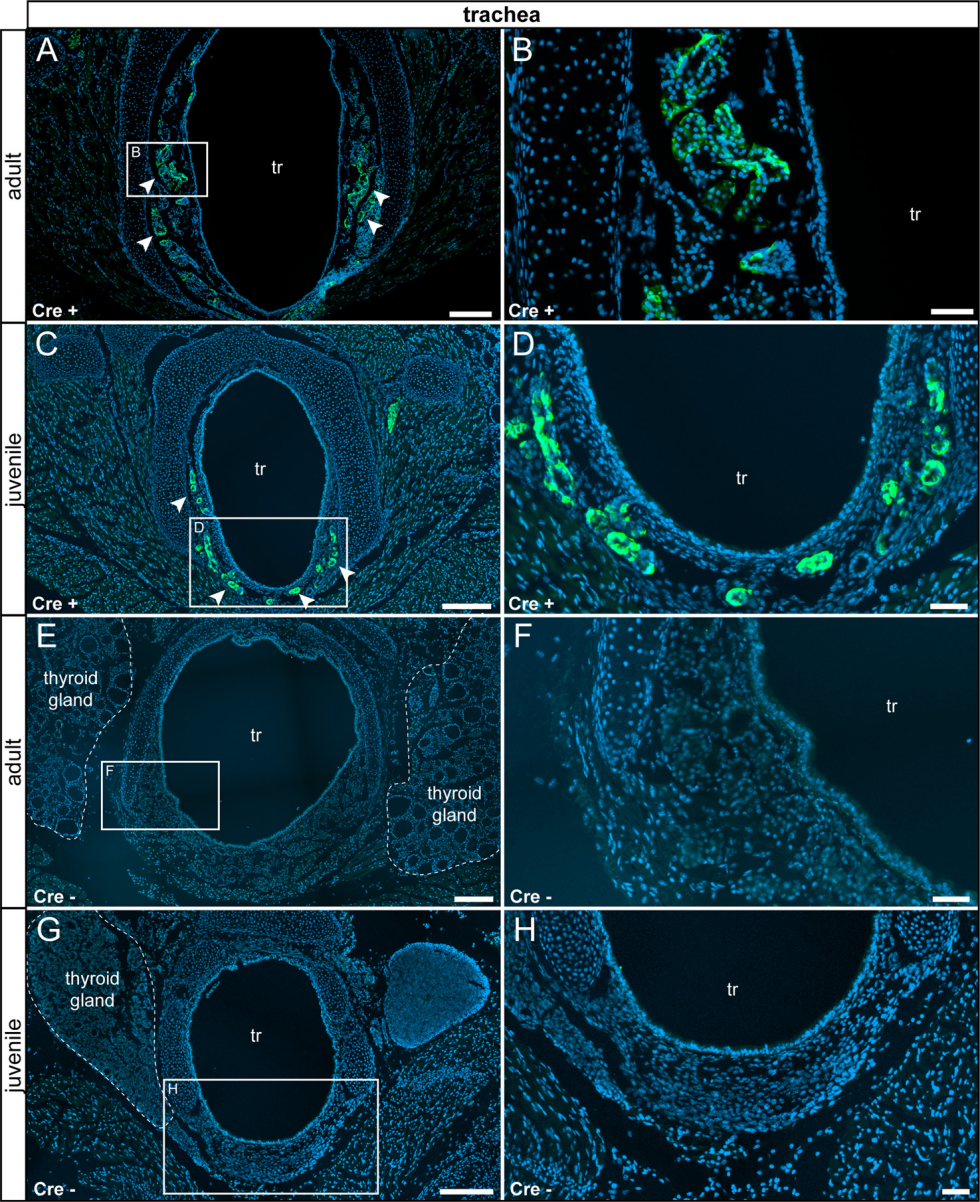
Detection of τGFP+ cells in a subset of mucous glands lining the trachea. (A) In adults, τGFP was observed in cells of the mucous glands (arrowheads) lining the trachea (tr). (B) Magnified image of area indicated in (A). (C) A similar pattern was picked up in juvenile Cre+ animals. (D) Magnified image of thearea indicated in (C). (E) No reporter gene expression was found in adult control animals (Cre-). (F) Magnified image of the area indicated in (E). (G) No reporter gene expression was found in the juvenile control trachea. (H) Magnified image of the area indicated in (G). tr: trachea. Thyroid gland indicated by stitched line. Nuclear stain in blue. Scale bars: 250 µm (A and C), 200 µm (E and G), 50 µm (B, D, F and H).

**Fig. 8:**
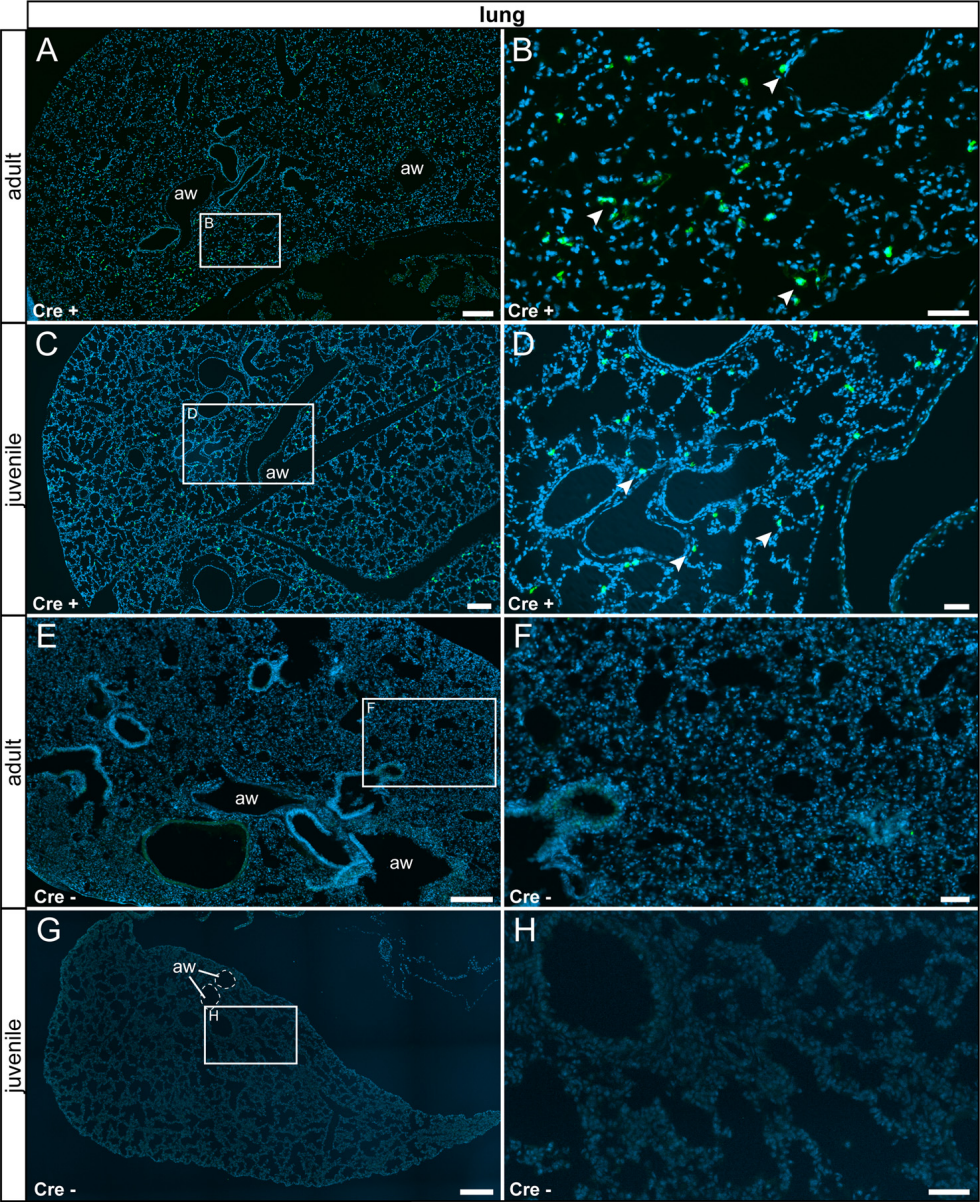
τGFP+ cells were observed in the lung of experimental animals. (A) A few scattered τGFP+ cells were detected in the adult Cre+ lung (arrowheads). (B) Magnified image of the area indicated in (A). (C) Juvenile animals showed the same staining pattern. (D) Magnified image of the area indicated in (C). (E) No τGFP+ cells were found in adult control tissue (Cre-). (F) Magnified image of area indicated in (E). (G) No reporter gene expression was observed in juvenile controls. (H) Magnified image of area indicated in (G). Nuclear stain in blue. Aw: airway. Scalebars: 200 µm (A and C), 500 µm (E and G), 50 µm (B, D, F and H).

We observed τGFP+ cells in a subset of myoepithelial cells in the glands lining the trachea in both adults and juveniles. We did not detect any reporter gene-expressing cells in control animals (Fig. 7). We observed scattered τGFP+ cells with a small cytoplasm in the lung of experimental but not control animals (Fig. 8).

**Fig. 9:**
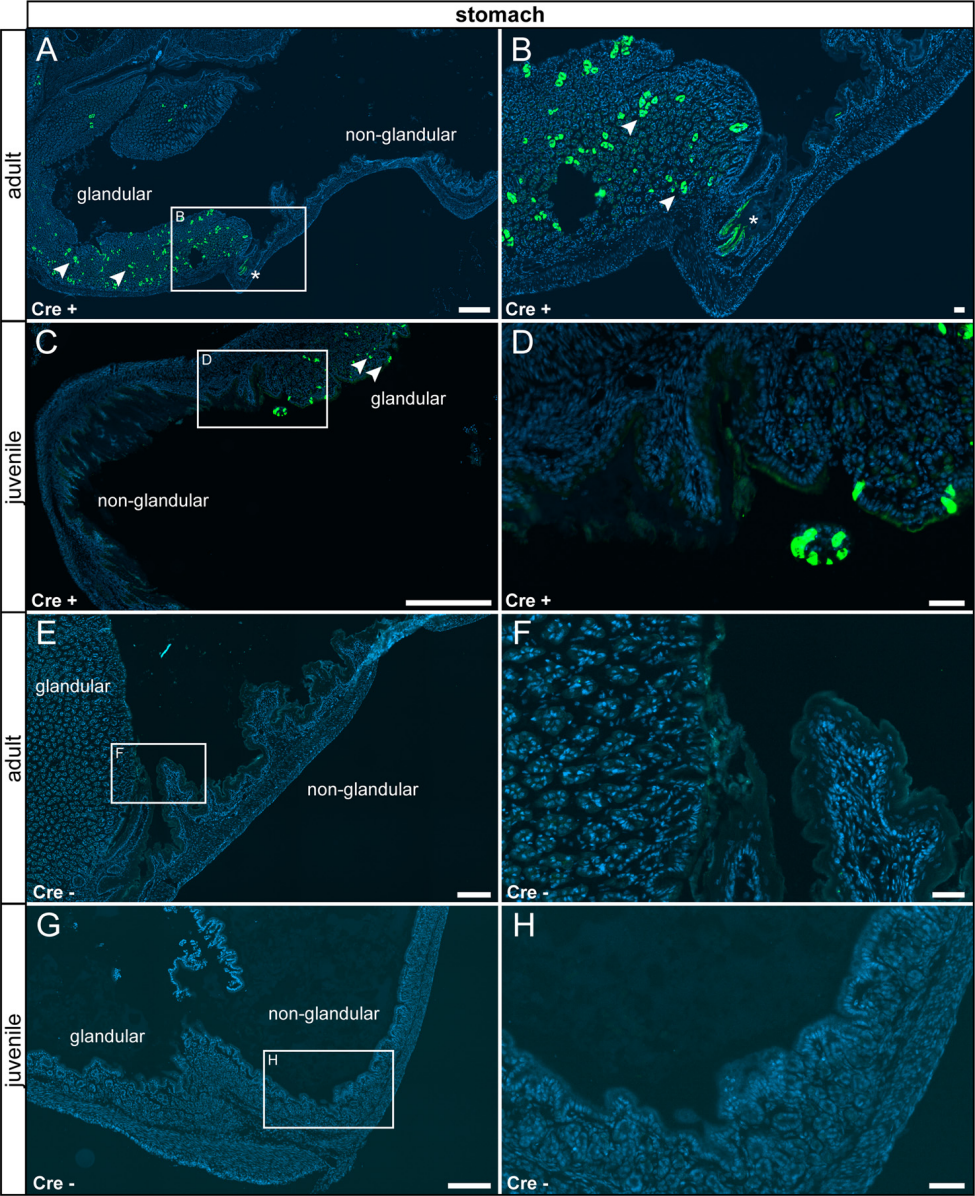
τGFP+ cells were observed in the glandular part of the stomach. (A) Few τGFP+ parietal (asterisk) and epithelial cells (arrowheads) were found in the stomach of adult reporter animals. (B) Magnified image of the area indicated in (A). (C) A similar pattern was picked up in juvenile Cre+ animals. (D) Magnified image of the area indicated in (C). (E) No reporter gene expression was observed in adult control tissue (Cre-). (F) Magnified image of area indicated in (E). (G) No reporter gene expression was observed in juvenile controls. (H) Magnified image of area indicated in (G). Nuclear stain in blue. Scalebars: 200 µm (A and C), 500 µm (E and G), 50 µm (B, D, F and H).

**Fig. 10:**
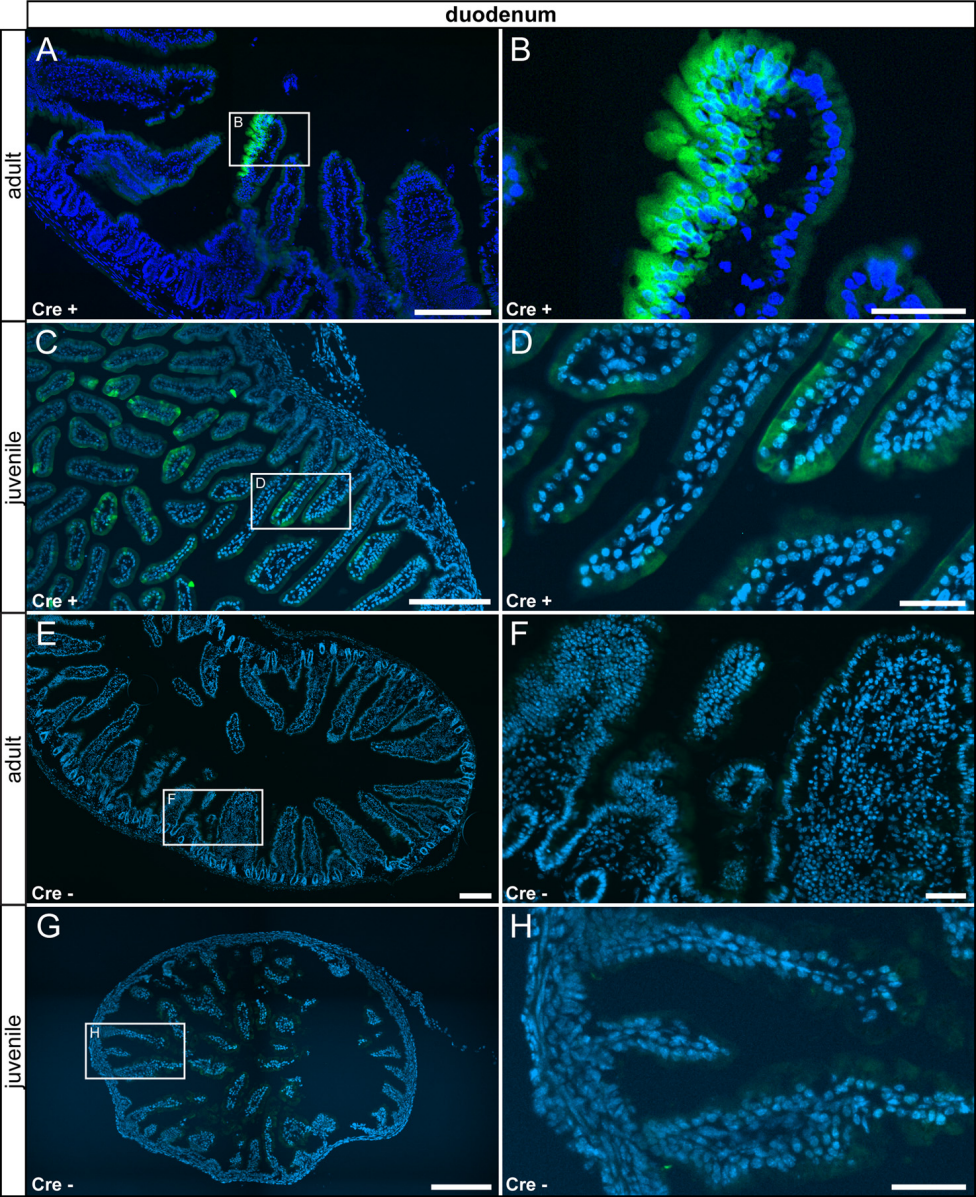
In the duodenum, a subset of epithelial cells expressed τGFP. (A) Low number of high prismatic epithelial cells were τGFP+ in the duodenum of adult Cre+ animals. (B) Magnified image of the area indicated in (A). (C) A similar expression pattern was observed in juvenile Cre+ animals. (D) Magnified image of area indicated in (C). (E) No reporter gene expression was observed in adult control tissue (Cre-). (F) Magnified image of the area indicated in (E). (G) No reporter gene expression was observed in juvenile controls. (H) Magnified image of area indicated in (G). Nuclear stain in blue. Scale bars: 200 µm (A, C, E and G), 50 µm (B, D, F and H).

**Fig. 11:**
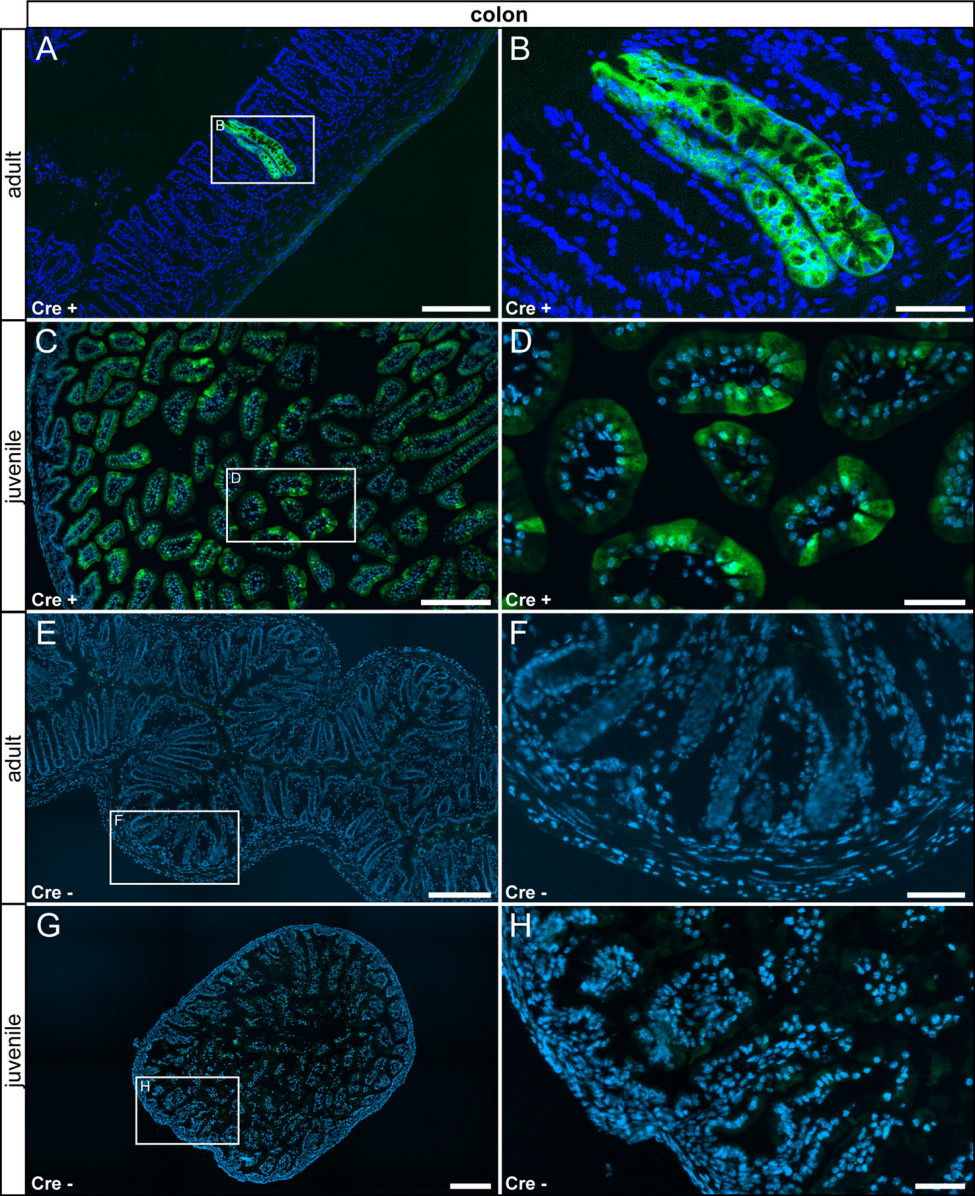
In the colon a subset of epithelial cells expressed τGFP. (A) Very few epithelial cells in the villi of the colon were τGFP+ in adult Cre+ animals. (B) Magnified image of the area indicated in (A). (C) The expression pattern in younger Cre+ animals was similar. (D) Magnified image of the area indicated in (C). (E) No reporter gene expression was observed in adult control tissue (Cre-). (F) Magnified image of the area indicated in (E). (G) No reporter gene expression was observed in juvenile controls. (H) Magnified image of the area indicated in (G). Nuclear stain in blue. Scale bars: 200 µm (A, C, E and G), 50 µm (B, D, F and H).

In the gastrointestinal tract, reporter gene expression was observed only in few sub compartments in specific cell types. In the stomach, we detected τGFP expression in the glandular part in columnar surface epithelial cells, in a subset of acid-secreting parietal cells and in high prismatic epithelial cells in the gastric glands. We did neither detect reporter gene expression in the non-glandular part nor in control tissue (Fig. 9). We also observed reporter gene expression in a subset of high prismatic epithelial cells in the duodenum (Fig. 10 A–D) and colon (Fig. 11 A–D). We did not detect reporter gene expression in intestinal tissue of controls (Fig. 10 E–H, Fig. 11 E–H).

**Fig. 12:**
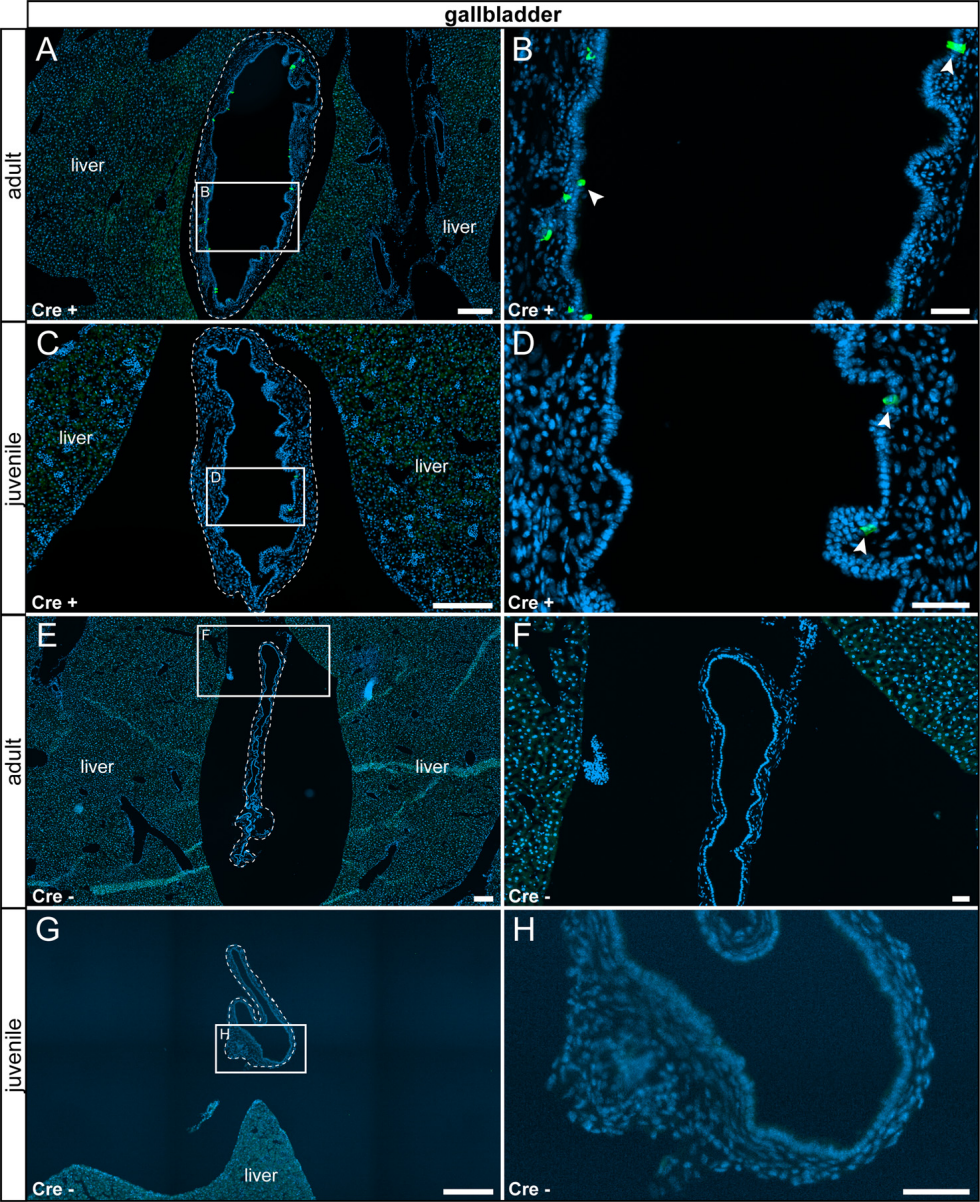
τGFP-expressing cells in the gallbladder (indicated by stitched line). (A) In the adult Cre+ gallbladder, a small subset of epithelial cells were τGFP+ (arrowheads). (B) Magnified image of the area indicated in (A). (C) A similar pattern was observed in juvenile Cre+ animals. (D) Magnified image of the area indicated in (C). (E) No reporter gene expression was found in adult control tissue (Cre-). (F) Magnified image of area indicated in (E). (G) No reporter gene expression was observed in juvenile controls. (H) Magnified image of area indicated in (G). Nuclear stain in blue. Scale bars: 200 µm (A, C, E and G), 50 µm (B, D, F and H).

**Fig. 13:**
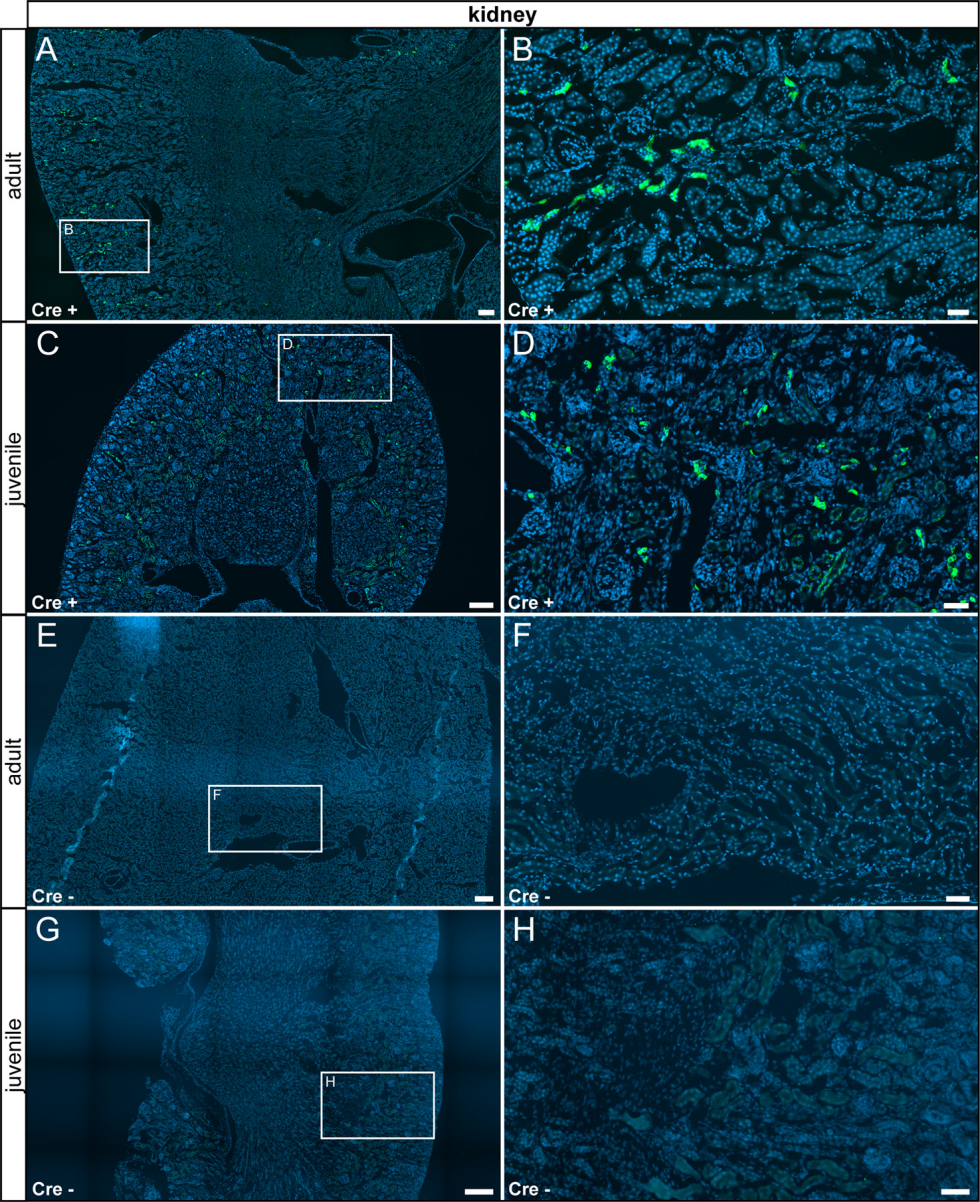
Few τGFP-expressing cells were found in the kidney. (A) In the adult Cre+ kidney, few cells in the cortex expressed τGFP. (B) Magnified image of area indicated in (A). (C) A similar pattern was found in Cre+ juveniles. (D) Magnified image of area indicated in (C). (E) No reporter gene expression was observed in adult control tissue (Cre-). (F) Magnified image of area indicated in (E). (G) No reporter gene expression was observed in juvenile controls. (H) Magnified image of area indicated in (G). Nuclear stain in blue. Scale bars: 200 µm (A, C, E and G), 50 µm (B, D, F and H).

**Fig. 14:**
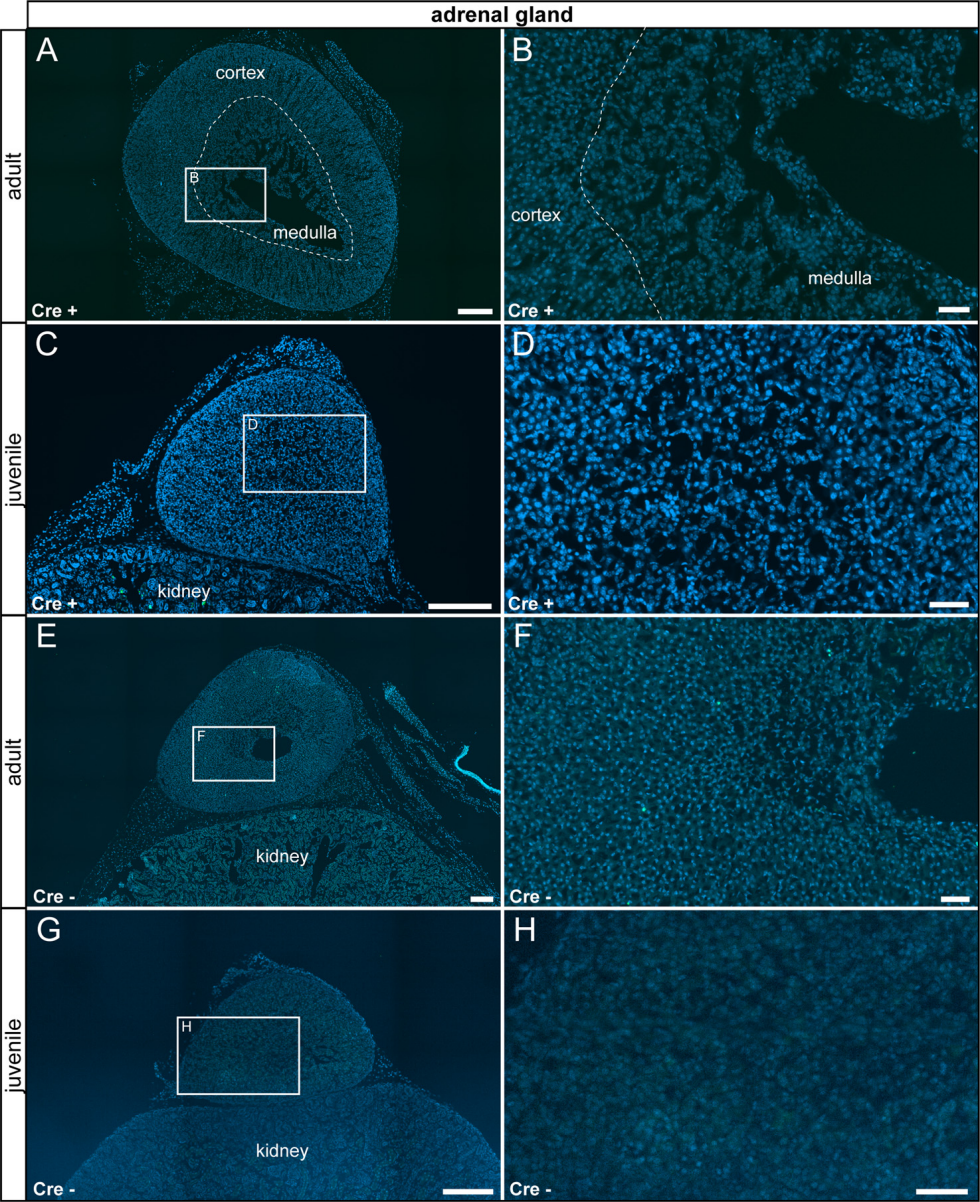
No τGFP-expression was observed in the adrenal gland of experimental (Cre+) and control (Cre-) animals. (A) to (H) No reporter gene expression was observed in the adrenal gland regardless of age and genotype. Nuclear stain in blue. Scale bars: 250 µm (A and C), 200 µm (E and G), 50 µm (B, D, F and H).

In the gallbladder, we detected sparse τGFP+ cells in epithelial cells of the mucosa, which were absent in control animals (Fig. 12). We detected reporter gene expression in the medulla and in the cortex of the kidney which were not part of the glomerulus. We did not detect any age-differences and no reporter gene expression was observed in control tissues (Fig. 13). We did not detect reporter gene expression in the adrenal gland at any stage analyzed (Fig. 14).

**Fig. 15:**
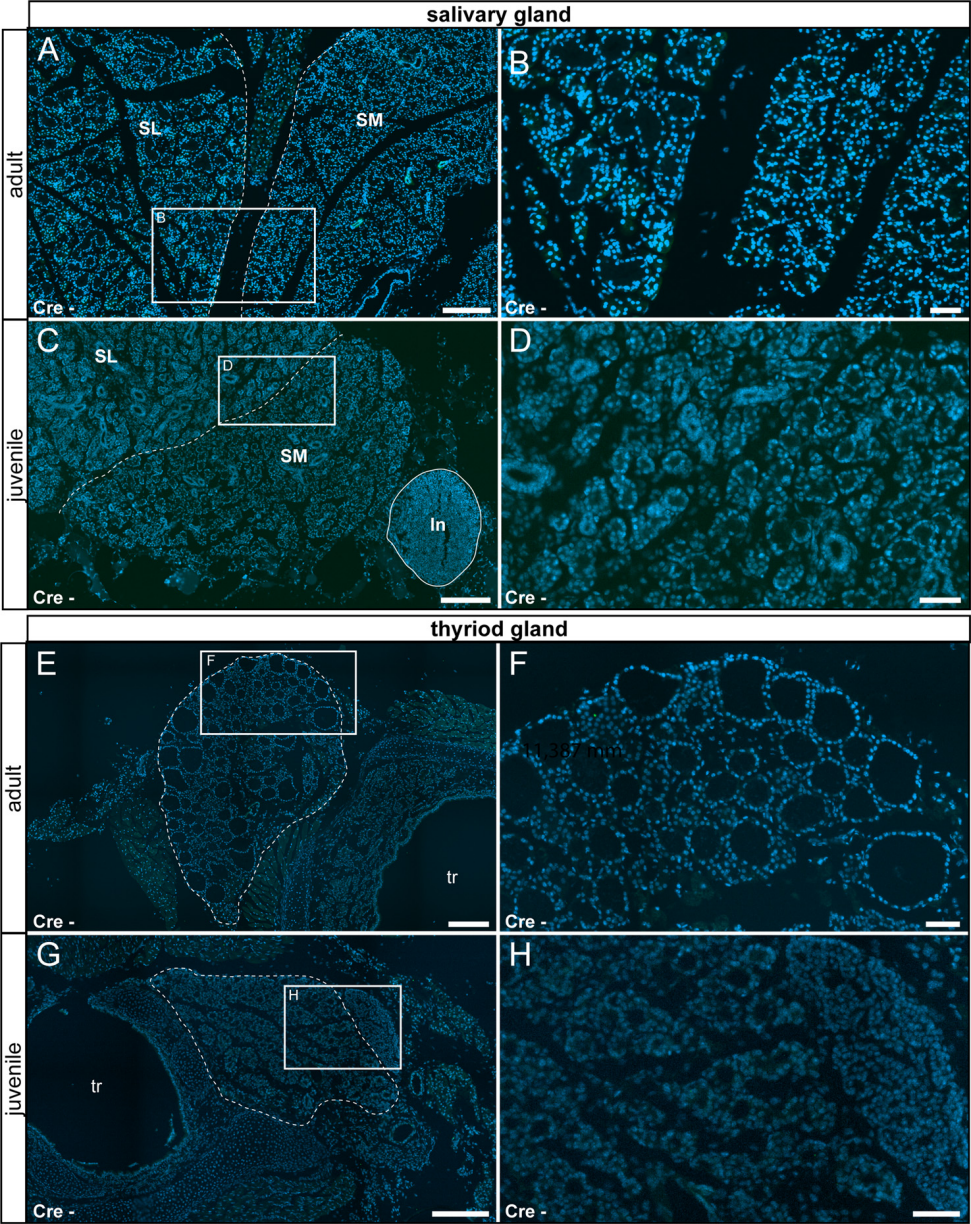
No reporter gene expression was observed in the salivary and thyroid glands of adult and juvenile control animals (Cre-). (A) Adult salivary gland was devoid of τGFP-expression. (B) Magnified image of the area indicated in (A). (C) Reporter gene expression was also absent in the juvenile salivary gland. Submandibular part (SM) and sublingual part (SL) are separated by a stitched line. Lymph node (ln) is indicated by solid line. (D) Magnified image of the area indicated in (C). (E) Adult thyroid gland (indicated by stitched line) was devoid of τGFP-expression. (F) Magnified image of area indicated in (E). (G) Absence of reporter gene expression in the juvenile thyroid gland. (H) Magnified image of area indicated in (G). tr: trachea. Nuclear stain in blue. Scale bars: 200 µm (A, C, E and G), 50 µm (B, D, F and H).

**Fig. 16:**
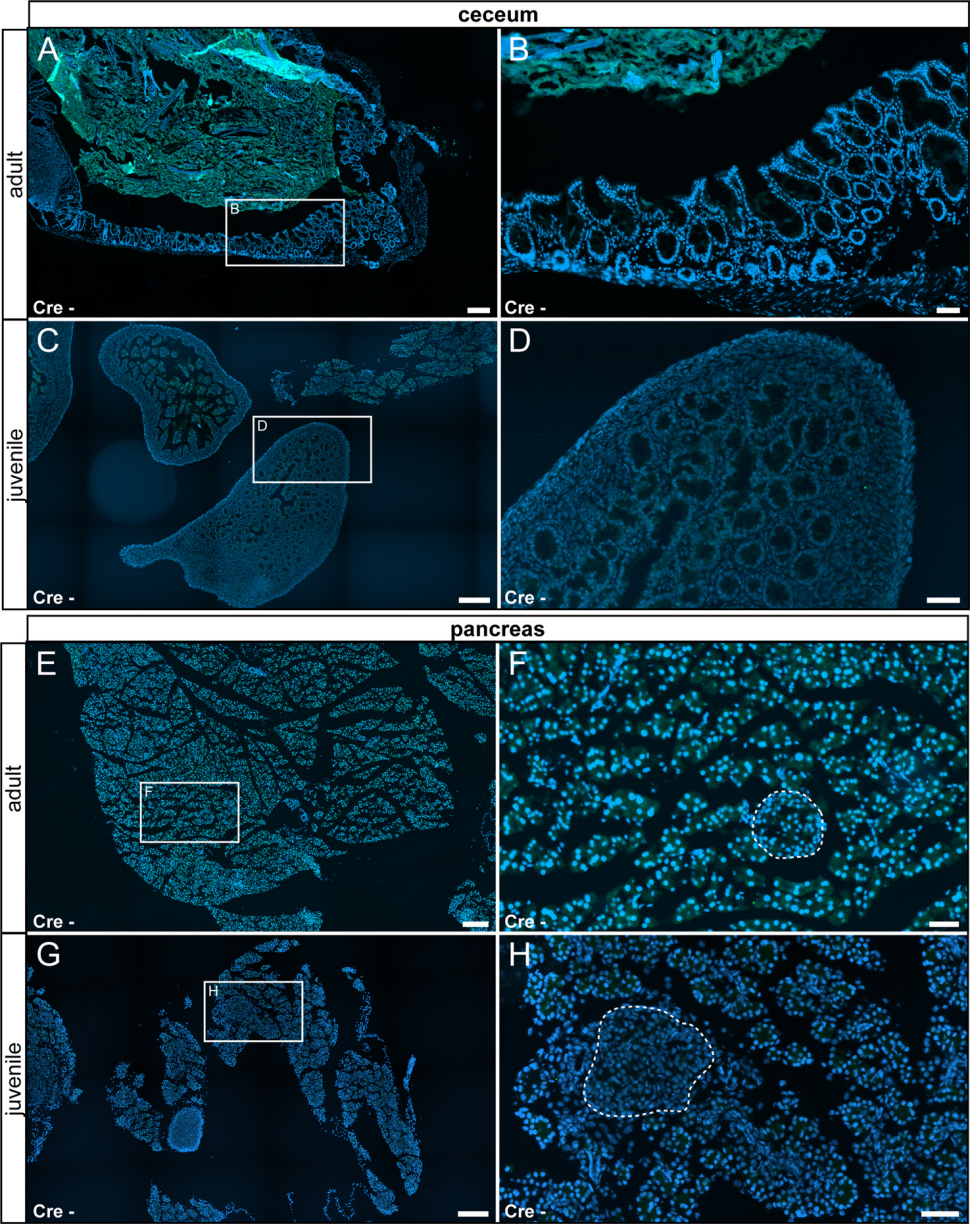
No reporter gene expression was observed in the cecum and pancreas of adult and juvenile control animals. (A) Adult cecum was devoid of τGFP-expression. (B) Magnified image of the area indicated in (A). (C) Absence of reporter gene expression in the juvenile cecum. (D) Magnified image of the area indicated in (C). (E) Adult pancreas was devoid of τGFP expression. (F) Magnified image of the area indicated in (E). (G) Absence of reporter gene expression in the juvenile pancreas. (H) Magnified image of the area indicated in (G). Islets of Langerhans are indicated by stitched line. Nuclear stain in blue. Scale bars: 200 µm (A, C, E and G), 50 µm (B, D, F and H).

**Fig. 17:**
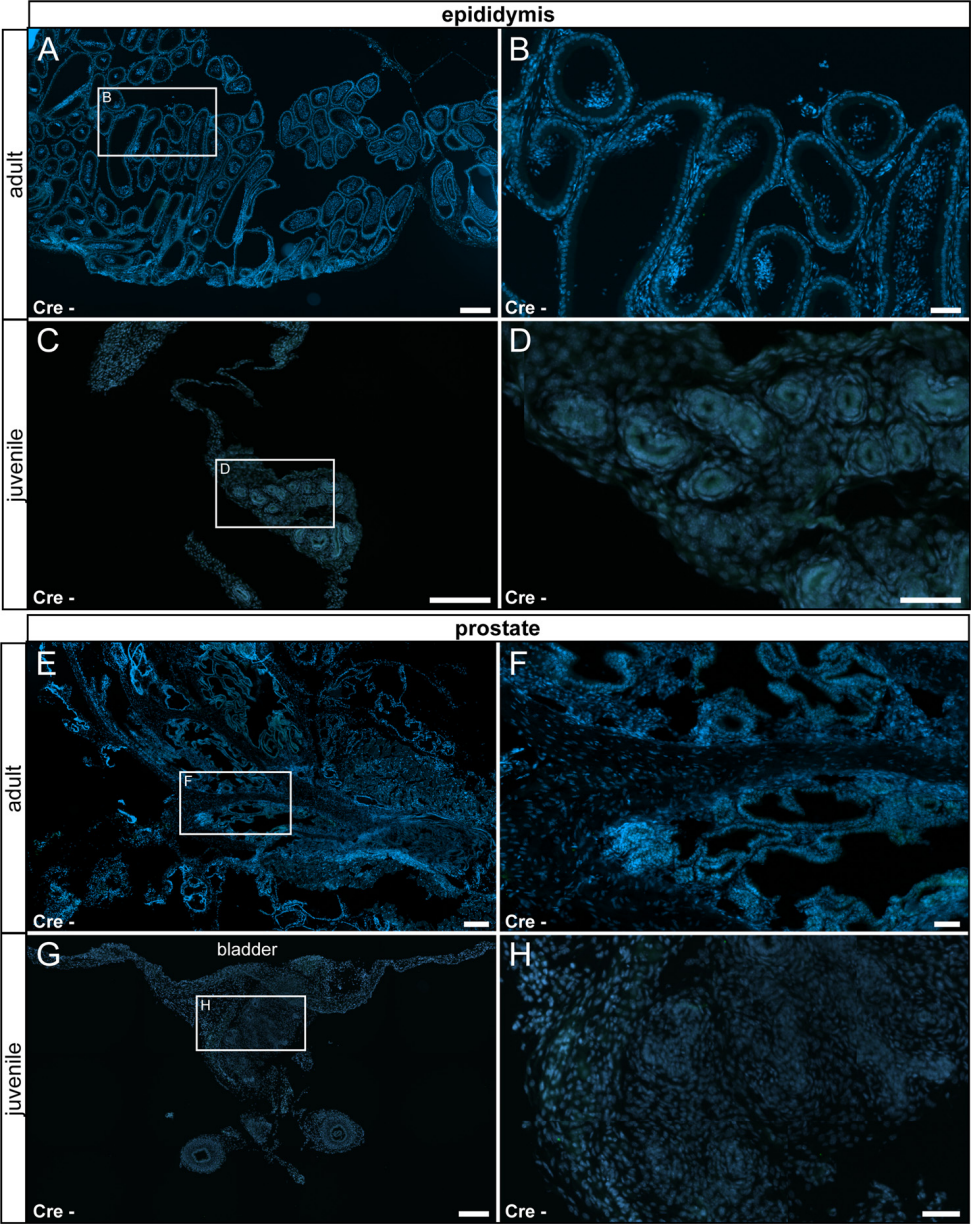
No reporter gene expression was observed in the epididymis and prostate of both adult and juvenile control animals. (A) In adult control animals, no τGFP-expression was observed in the epididymis. (B) Magnified image of area indicated in (A). (C) Absence of reporter gene expression in juvenile animals. (D) Magnified image of the area indicated in (C). (E) No reporter gene expression was found in the adult prostate. (F) Magnified image of area indicated in (E). (G) Absence of reporter gene expression in the juvenile prostate. (H) Magnified image of the area indicated in (G). Nuclear stain in blue. Scale bars: 200 µm (A, C, E and G), 50 µm (B, D, F and H).

**Fig. 18:**
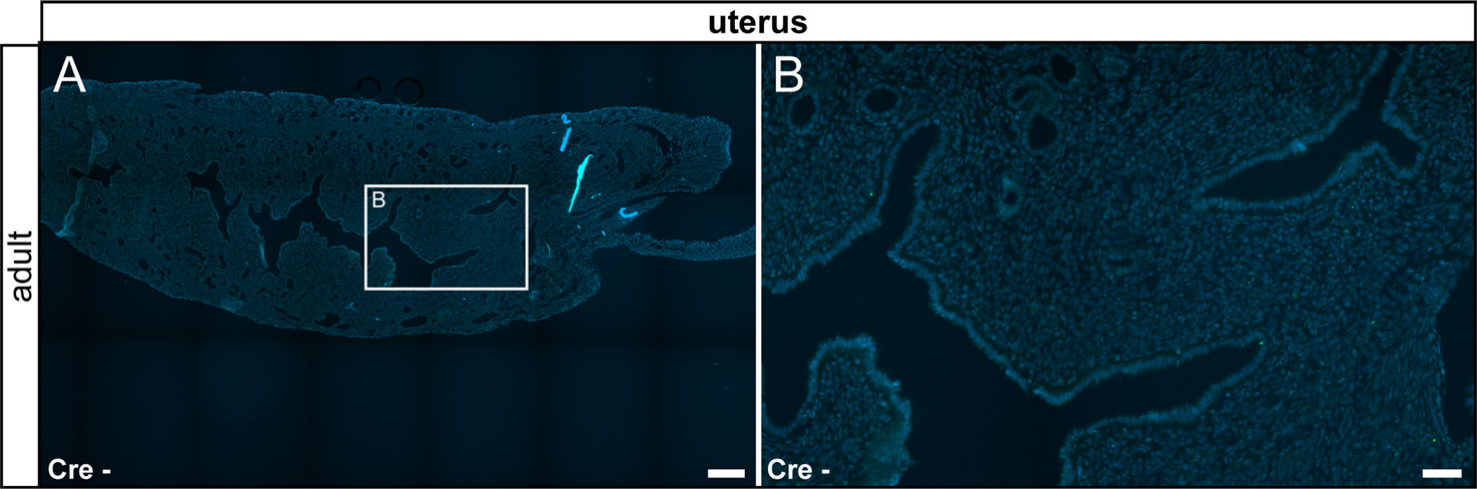
No fluorescent signal was observed in the adult uterus of control animals. Nuclear stain in blue. Scale bars: 200 µm (A and C), 50 µm (B and D).

In our original research article (Wartenberg et al. *Cell Calcium* 100:102481, 2021), we described TRPV6 expression in the salivary gland, the thyroid gland, the cecum, the pancreas, the epididymis, the prostate and the uterus by fluorescent labeling in TRPV6 reporter animals and confirmed by mass spectrometry. Here we corroborate the specificity of the fluorescent labeling by documenting the absence of signal in tissue prepared from reporter animals (Figs. 15 – 18).

## 2 Experimental Design, Materials and Methods

TRPV6-IRES-Cre (TRPV6-IC) animals were generated as described [2] and bred to enhanced-ROSA26-τGFP (eR26-τGFP) reporter mice [3] to generate TRPV6-IC/eR26-τGFP animals (Cre+). Cre-/eR26-τGFP reporter animals were used as controls. Mice were kept under a standard light/dark cycle and food and water *ad libitum*.

For organ collection, adult (8–12 weeks) and juvenile (postnatal day 5) Cre+ and Cre- animals of both sexes were used. Animals were anesthetized with a mix of ketamine and xylazine and transcardially perfused with PBS followed by ice-cold 4% paraformaldehyde (PFA) in PBS. Organs were dissected as described elsewhere [4] and post-fixed in ice-cold PFA for 3 h. After post-fixation, organs were cryoprotected in 18% sucrose in PBS over night at 4 °C. The nose was decalcified in 0.5 M EDTA for three to four days at 4 °C prior to cryoprotection. Organs were frozen in freezing medium (OCT, Leica) and stored at −80 °C until usage.

Representative sections of each organ were prepared with 10 µm thickness (brain and pituitary 14 µm) and stored at −80 °C until staining. For immunofluorescence, sections were washed 3 times in PBS for rehydration at room temperature (RT) and then incubated in blocking solution (10% normal donkey serum (Jackson Immuno Research), 3% bovine serum albumin (Sigma) and 0.3% TX-100 (Roth)) for 1 h at RT. All antibodies were diluted in PBS-Carrageenan (Sigma). After blocking, sections were incubated in primary antibody (rabbit anti-GFP, 1:1000, Thermo Fischer) over night at 4 °C. On the following day, sections were washed three times for five minutes each in PBST (0.05% Tween-20 in PBS) and incubated for two hours at RT in secondary antibody (donkey anti-rabbit biotinylated, 1:500, Jackson Immuno Research) followed by three washes in PBST for five minutes each and one hour incubation in Cy5 conjugated streptavidin (1:500, Jackson Immuno Research) at RT. Sections were then washed three times in PBST and incubated in bisbenzimide (1:10000, Sigma) for 10 min at RT. Sections were coverslipped in Fluoromount G (Southern Biontech) and stored at 4 °C until imaging.

Imaging was performed with the AxioScan Slidescanner (Zeiss) using the ZenBlue software.

## Ethics Statements

All animal experiments were performed according to the Guide for the Care and Use of Laboratory Animals published by the United States National Institutes of Health and were approved by the local ethics committee and Kreispolizeibehörde des Saarpfalz-Kreises, Germany (approval number GB3-2.4.7.1-Boe, 08.01.2021). Adult (8 – 12 weeks) and juvenile (postnatal day 5) animals of both sexes were used.

## CRediT Author Statement

**Philipp Wartenberg:** Methodology, Software, Data curation. Writing. **Petra Weissgerber, Ulrich Boehm:** Conceptualization, Supervision, Original draft preparation. **Kai Busch, Femke Lux:** Methodology, Visualization. **Claudia Fecher-Trost, Gabriela Krasteva-Christ, Veit Flockerzi:** Reviewing and Editing. Data interpretation: all authors.

## Declaration of Competing Interest

The authors declare that they have no known competing financial interests or personal relationships that could have appeared to influence the work reported in this paper.

## Data Availability

Wartenberg et al_Images (Original data) (Dryad).
